# Biomechanical comparison of anterior axis-atlanto-occipital transarticular fixation and anterior atlantoaxial transarticular fixation after odontoidectomy: A finite element analysis

**DOI:** 10.3389/fbioe.2023.1067049

**Published:** 2023-03-07

**Authors:** Yuzhao Lu, Gai Hang, Yu Feng, Bo Chen, Shenghui Ma, Yang Wang, Tianhao Xie

**Affiliations:** ^1^ The First Affiliated Hospital of Nanchang University, Nanchang University, Nanchang, Jiangxi, China; ^2^ School of Medicine, Wuhan University of Science and Technology, Wuhan, Hubei, China; ^3^ Ruijin Hospital, School of Medicine, Shanghai Jiao Tong University, Shanghai, China; ^4^ Beijing Chaoyang Hospital, Capital Medical University, Beijing, China; ^5^ General Hospital of Central Theater Command, Wuhan, Hubei, China

**Keywords:** anterior axis-atlanto-occipital transarticular fixation, anterior atlantoaxial transarticular fixation, finite element analysis, odontoidectomy, biomechanics

## Abstract

**Background:** Anterior axis-atlanto-occipital transarticular fixation (AAOF) and anterior atlanto-axial transarticular fixation (AAF) are two common anterior screw fixation techniques after odontoidectomy, but the biomechanical discrepancies between them remain unknown.

**Objectives:** To investigate the biomechanical properties of craniovertebral junction (CVJ) after odontoidectomy, with AAOF or AAF.

**Methods:** A validated finite element model of the intact occipital-cervical spine (from occiput to T1) was modified to investigate biomechanical changes, resulting from odontoidectomy, odontoidectomy with AAOF, and odontoidectomy with AAF.

**Results:** After odontoidectomy, the range of motion (ROM) at C1-C2 increased in all loading directions, and the ROM at the Occiput-C1 elevated by 66.2%, 57.5%, and 41.7% in extension, lateral bending, and torsion, respectively. For fixation models, the ROM at the C1-C2 junction was observably reduced after odontoidectomy with AAOF and odontoidectomy with AAF. In addition, at the Occiput-C1, the ROM of odontoidectomy with AAOF model was notably lower than the normal model in extension (94.9%), flexion (97.6%), lateral bending (91.8%), and torsion (96.4%). But compared with the normal model, in the odontoidectomy with AAF model, the ROM of the Occiput-C1 increased by 52.2%, −0.1%, 92.1%, and 34.2% in extension, lateral bending, and torsion, respectively. Moreover, there were no distinctive differences in the stress at the screw-bone interface or the C2-C3 intervertebral disc between the two fixation systems.

**Conclusion:** AAOF can maintain CVJ stability at the Occiput-C1 after odontoidectomy, but AAF cannot. Thus, for patients with pre-existing atlanto-occipital joint instability, AAOF is more suitable than AAF in the choice of anterior fixation techniques.

## Introduction

Pathological disorders in the craniovertebral junction (CVJ) including non-reducible ventral bony compression, rheumatoid inflammation, tumoral lesions, tubercular diseases, and metabolic-related diseases which usually lead to severe ventral compression (Yang and Gao, 1999; Kerschbaumer et al., 2000; Tubbs et al., 2016). To alleviate the compression of the brain stem or spinal cord, odontoidectomy (transoral or transnasal), is commonly used as an effective decompression approach. However, clinical and *in vitro* studies have indicated that this technique may give rise to progressive craniovertebral instability (Dickman et al., 1992; Dickman et al., 1995).

Posterior fixation is commonly performed as a subsequent operation following odontoidectomy to address CVJ instability (Grob et al., 1992). However, when posterior fixation is unavailable in some clinical cases, anterior fixation technology represented by anterior atlantoaxial transarticular fixation (AAF) has been developed as an alternative (Lu et al., 1998). Compared with posterior fixation, AAF is less likely to damage the vertebral artery and can be performed during odontoidectomy to avoid an additional operation (Sen et al., 2005). Besides, it was demonstrated that AAF was able to efficiently maintain the stability of C1-C2 after odontoidectomy (Magerl and Seemann, 1987). However, the stability at Occiput-C1 was also damaged after odontoidectomy, owing to the destroyed important structures, such as the anterior atlantooccipital membrane, the apical ligament, and the alar ligament which were closely related to the stability of Occiput-C1 (Dickman et al., 1992; Dickman et al., 1995). Moreover, the stability of Occiput-C1 is also crucial for the human body (Dvorak et al., 2003a), especially for patients with instability of Occiput-C1 for the pathology itself.

Anterior axis-atlanto-occipital transarticular fixation (AAOF) is another anterior fixation technique that can retain stability from the occiput to the axis (Dvorak et al., 2003a). Nevertheless, few biomechanical studies have examined whether AAOF can maintain CVJ stability (from the occiput to the axis) after odontoidectomy, and no study has examined the biomechanical difference between AAOF and AAF under odontoidectomy. Thus, our study aimed to investigate the biomechanical properties of AAOF after odontoidectomy and its differences from AAF after odontoidectomy.

## Materials and methods

**FIGURE 1:**
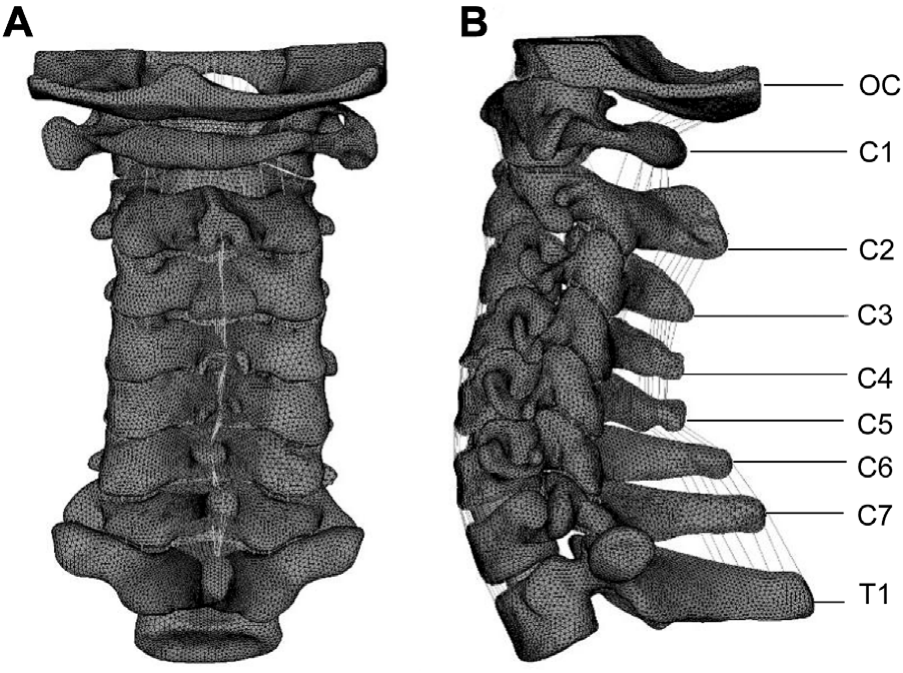
Posterior **(A)** and lateral **(B)** views of the finite element model of the whole cervical vertebra (OC-T1). Reproduced from the ref. (Xie et al., 2021), Copyright (2021), with permission from Elsevier.

A previously reported nonlinear three-dimensional finite element model (FEM) of a normal whole cervical spine (C0-T1) was used in this study, as shown in Figure 1 (Xie et al., 2021). To validate the intact model, the predicted kinematic results were analyzed and compared with those reported in the literature (Penning, 1978; Penning and Wilmink, 1987; Lind et al., 1989; Panjabi et al., 1998; Ito et al., 2004; Ishii et al., 2006). The validated results of our whole cervical spine were presented in Supplementary Figure S1 (Xie et al., 2021). A CT scan of a healthy volunteer (34 years old, male, height 175 cm, body mass 70 kg) was used to build this finite element model. The detailed values for various materials and properties are the most commonly used values obtained from the literature (Xie et al., 2013; Xie et al., 2021). The material properties are detailed in Supplementary Tables S1–3 (Xie et al., 2021). And informed consent was obtained from the volunteer. The entire study was compliant with the Helsinki Declaration.

**FIGURE 2:**
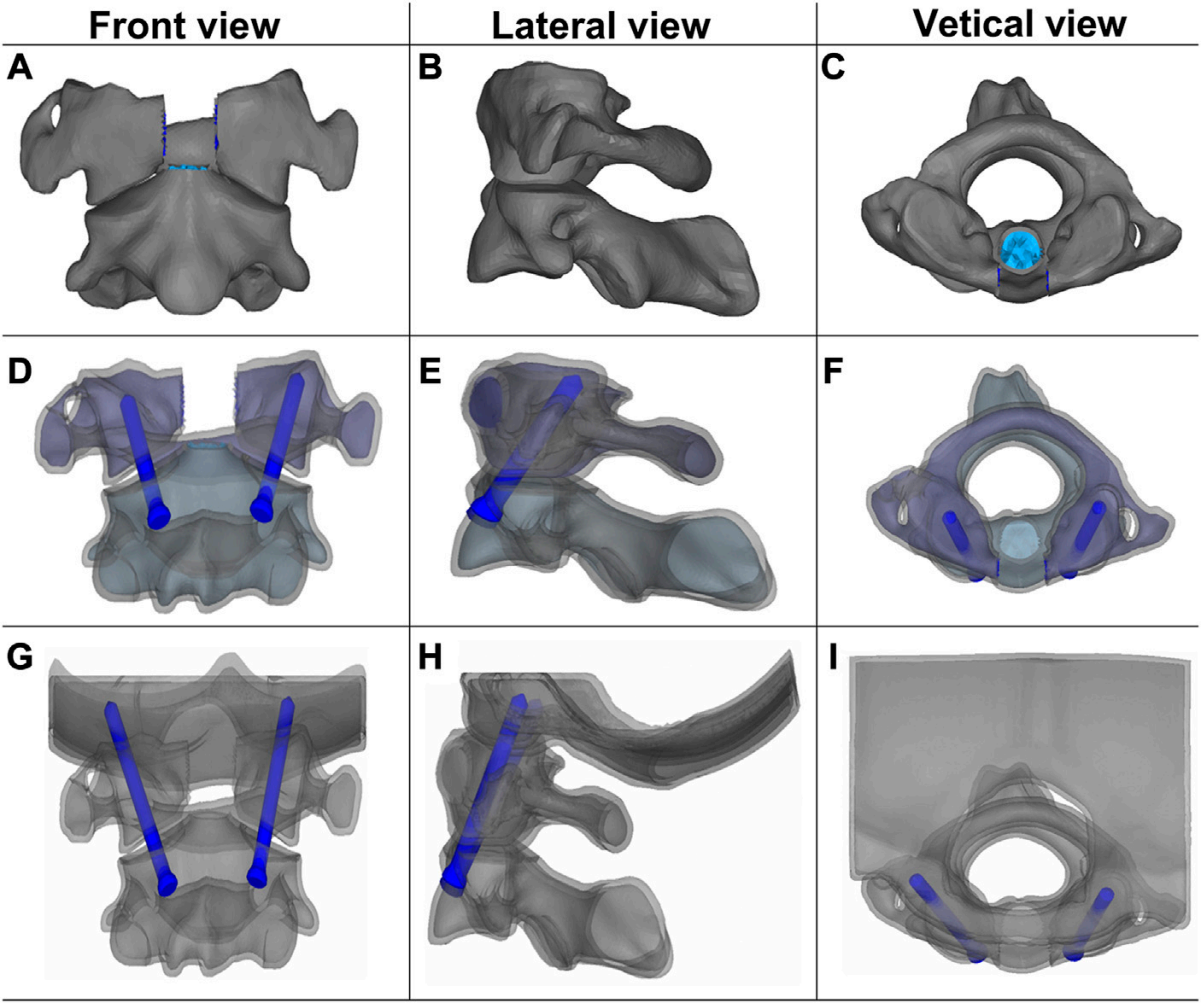
Front, lateral, and vertical views of the three surgical finite element models: Odontoidectomy **(A–C)**, AAF **(D–F)**, and AAOF **(G–I)** models. AAOF, anterior axis-atlanto-occipital transarticular fixation; AAF, anterior atlantoaxial transarticular fixation. **(A)** was reproduced from the ref. (Xie et al., 2021), Copyright (2021), with permission from Elsevier.

Based on the validated cervical spine model, odontoidectomy model, odontoidectomy with AAF model, and odontoidectomy with AAOF model were simulated in this study. The anatomical changes of the three surgical models were constructed according to the real surgical procedures reported in the literature (Figures 2A-I) (Dickman et al., 1995; Lu et al., 1998; Dvorak et al., 2003a; Xie et al., 2021). All models in our study, including the original complete spine model, were analyzed by the finite element software Abaqus 6.12.-1 (SIMULIA Inc, Providence, RI, United States). The selection of mesh type and mesh order in this analysis differs depending on the balance between the computational accuracy and modeling cost. For the bony structure of the cervical spine, C3D4 (linear tetrahedral) and C3D6 (linear trihedral) meshes were used for meshing. For the intervertebral discs, C3D8R (linear hexahedral) with hourglass control was used for modeling. All nodes on the lower surface of the T1 vertebral body were constrained to be fixed in all directions as boundary conditions. The vertebral body and ligaments were connected by shared nodes, and so are the different components of internal fixation. And for the intervertebral disc and the cortical bone, ties were used. A nonlinear surface-to-surface contact was used to simulate the interactions between vertebral joints. Screws are connected to the bones using an embedded setting. The elastic modulus of the medical titanium used for the construction of the AAF and AAOF screws was 110,000 MPa and Poisson’s ratio was 0.30 (Rohlmann et al., 2007).

1. The Odontoidectomy model: The C1 anterior arch, C1-C2 anterior longitudinal ligament, anterior atlantooccipital membrane, odontoid, and the ligaments connecting to the odontoid (transverse ligament, alar ligament, apical ligament, and cruciate ligament vertical portion) were removed from the intact C0-T1 model (Dickman et al., 1995).
2. The AAF model (Odontoidectomy with AAF): The anterior atlantoaxial transarticular screw was added to the odontoidectomy model by using Solidworks/UGS software. And the screw insertion point was at the junction of the lateral edge of the C2 vertebral body to 4 mm above the inferior edge of the C2 anterior arch. The screw diameter and length were 3.5 mm and 15 mm, respectively (Lu et al., 1998).
3. The AAOF model (Odontoidectomy with AAOF): The anterior occiput-to-axis screw was drawn using Solidworks/UGS software and added to the odontoidectomy model according to the literature. The caudal of the center of the medial third of the C2 lateral mass articulation was the insertion point for AAOF. From the insertion point, the screw was placed obliquely in an outward, superior, and posterior direction, proceeding through the pivotal vertebral body, atlantoaxial joint, lateral atlantoaxial block, atlantooccipital joint, and successively anchored to the occipital condyle. To avoid injuring the sublingual neural tube and the vertebral artery, the screw was placed with a posterior angle of 25° and an external angle of 15° so that it connected with the posterior third of the occipital condyle. The screw diameter and length were 3.5 mm and 28 mm, respectively (Dvorak et al., 2003a).

Static analysis was conducted by imposing pure moments (sagittal, transverse, and frontal planes) of 1.5 Nm and a 50 N of compressive follower superior on the superior surfaces of occipital bone while the inferior surface of the T1 vertebra was rigidly fixed. ROM (°) values at Occiput-C1 and C1-C2 junctions of the four models including the normal model, odontoidectomy model, AAF model, and AAOF model under extension, flexion, lateral bending, and torsion conditions were measured. Accordingly, the percentage changes (%) of ROM for odontoidectomy model, AAF model, and AAOF model relative to the normal model were calculated ((surgical model-normal model)/normal model) and recorded to one decimal point. Besides, the maximum von Mises stress (MPa) on the implant and C2-C3 disc in the two anterior fixation models under extension, flexion, left bending, right bending, left torsion, and right torsion was also measured. The values of ROM changes and maximum stress mentioned above are shown in the histograms plotted by Prism 8.0 (GraphPad Software, United States).

## Results

### The ROM of the surgical models

**FIGURE 3:**
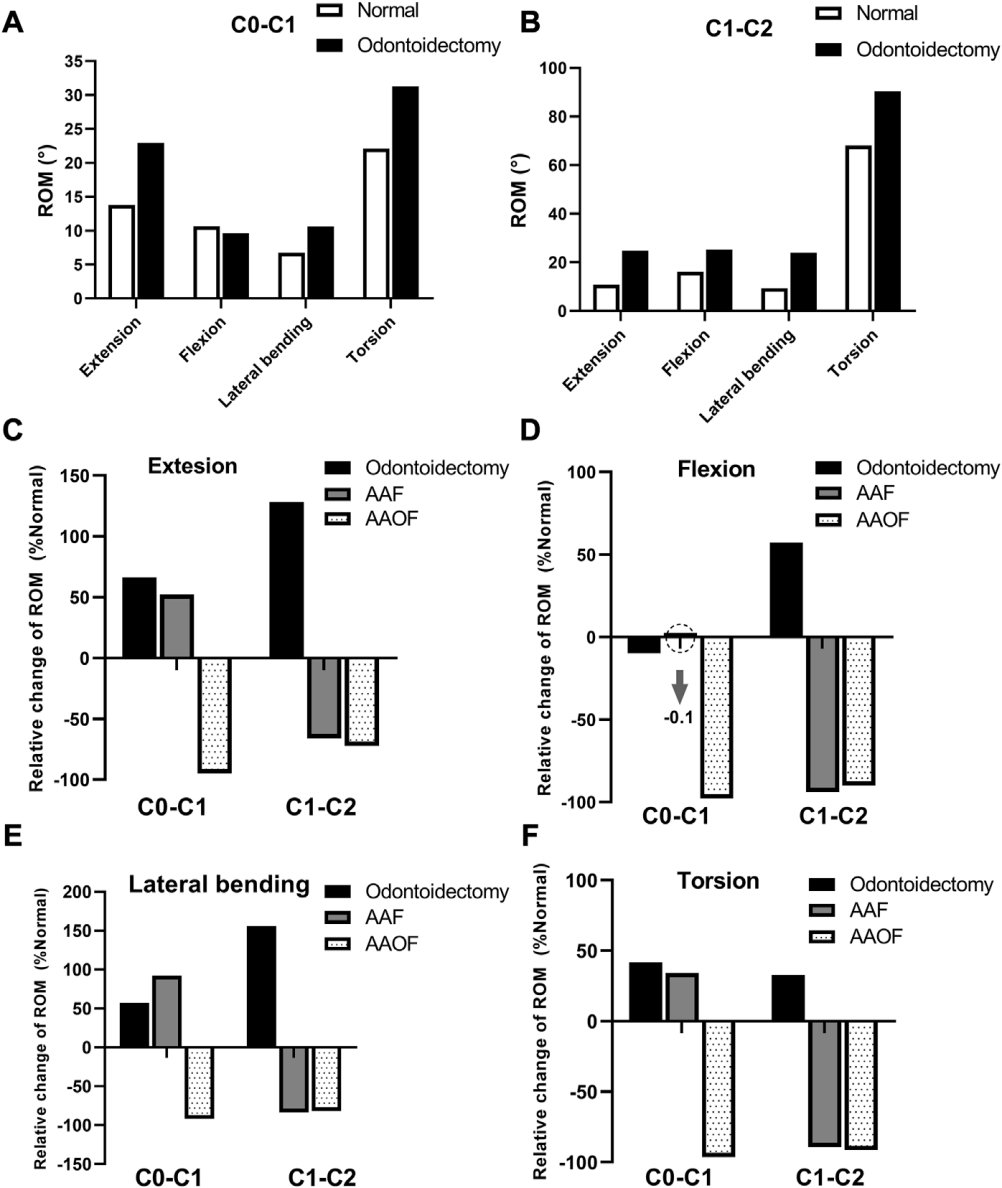
The changes of ROM (°) of the three surgical models compared to the normal model at Occiput-C1 and C1-C2 junctions. ROM = range of motion **(A, B)** presented the ROM (°) of odontoidectomy model and normal model at Occiput-C1 and C1-C2 junctions, respectively. And in **(C–F)**, the percentage change of ROMs for the odontoidectomy, AAF, and AAOF models relative to the normal model were shown.

Compared with the normal model, the ROM of odontoidectomy model at the Occiput-C1 junction increased by 66.2%, 57.5%, 41.7%, and −9.8% under loads of extension, lateral bending, torsion, flexion, respectively (Figure 3); the ROM at C1-C2 junction increased by 128.1%, 57.2%, 155.8%, and 32.8% in extension, flexion, lateral bending, and torsion, respectively (Figure 3).

At the C1-C2 junction, there was no significant difference between the two anterior fixation surgical models in the ROM changes (%) compared with the normal model (Figures 3C–F). In the AAF model, the ROM of the C1-C2 junction decreased by 66.1%, 93.8%, 83.5%, and 89.2% in extension, flexion, lateral bending, and torsion, respectively. In the AAOF model, the ROM of the C1-C2 junction decreased by 72.3%, 89.9%, 81.9%, and 91.5% in extension, flexion, lateral bending, and torsion, respectively.

But at the Occiput-C1 junction, the results revealed disparate trends of ROM changes (%) in the two anterior fixation surgical models (Figures 3C–F). In the AAF model, the ROM of the Occiput-C1 junction increased by 52.2%, −0.1%, 92.1%, and 34.2% in extension, flexion, lateral bending, and torsion, respectively. In the AAOF model, the ROM of the Occiput-C1 junction decreased by 94.9%, 97.6%, 91.8%, and 96.4% in extension, flexion, lateral bending, and torsion, respectively.

### The maximum von Mises stress on the implant and C2-C3

**FIGURE 4:**
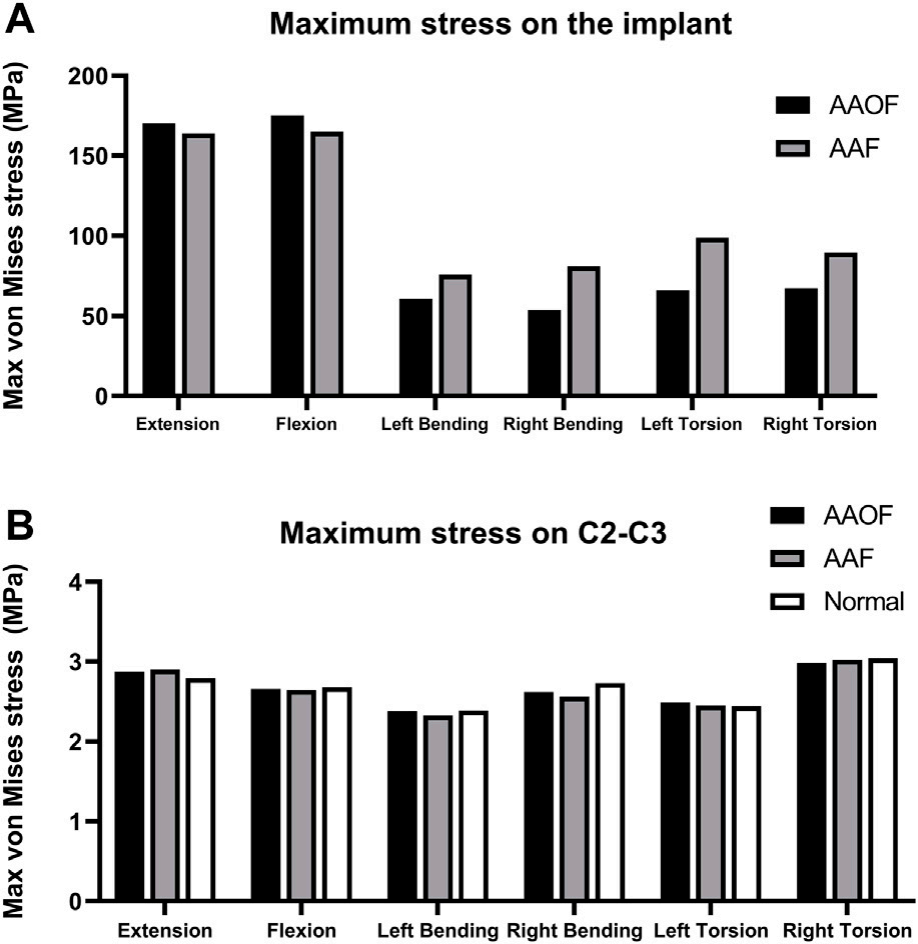
The maximum **v**on Mises stress (Mpa) on the implants **(A)** and C2-C3 intervertebral discs **(B)** for AAF and AAOF models. AAOF, anterior axis-atlanto-occipital transarticular fixation; AAF, anterior atlantoaxial transarticular fixation.

Figure 4 and Supplementary Figures S2–6 presented the maximum von Mises stress on the screw and C2-C3 of AAF and AAOF models. The maximum stress of AAOF implantation under extension and flexion (170.2 MPa and 175.3MPa, respectively) was greater than that of AAF (164.1 MPa and 165MPa, respectively) (Figure 4 A; Supplementary Figures S2, 3). However, under left bending, right bending left torsion, and right torsion, the maximum stress of AAOF implantation (60.73Mpa, 53.69Mpa, 65.85Mpa, and 53.69Mpa, respectively) was less than that of AAF (75.92Mpa, 81.11Mpa, 98.76Mpa, and 89.78Mpa, respectively) (Figure 4A). There is no obvious difference in the maximum von Mises stresses on the C2-C3 junction in extension, flexion, left bending, right bending, left torsion, and right torsion (<1 MPa) among the AAF model, the AAOF model, and the normal model (Figure 4B; Supplementary Figures S4–6).

## Discussion

In this study, a whole cervical spine model established and validated in our previous research (Xie et al., 2021) was used to investigate the biomechanics of surgical models and fixation techniques. Compared with the past studies which only constructed models of the upper cervical segment (Zhang et al., 2016; Chun et al., 2018), this study is more consistent with the integrity of the cervical spine.

### The stability of CVJ after odontoidectomy

Odontoidectomy was a standard surgical approach for anterior decompression since Kanavel et al. first used it in clinical practice (Kanavel, 1917). Considering the potential complications, the use of this operation is gradually decreasing with the introduction of posterior reduction techniques by Goel et al. (Goel, 2005). However, transoral odontoidectomy is still the first choice when posterior reduction techniques are not available. And Govindasamy et al. think the application scope of transoral odontoidectomy should be broader than the current use (Govindasamy et al., 2020).

The instability of the CVJ is one of the main complications after odontoidectomy. It is reported that more than two-thirds of patients experience significant spinal instability after odontoidectomy (Dickman et al., 1992). This operation may lead to significant biomechanical changes in the CVJ due to the excision of the odontoid process, anterior arch of the atlas, pterygoid ligament, odontoid apex ligament, transverse ligament, and other critical components attached to these bony structures. Dickman et al. found that odontoidectomy led to significant changes in ROM at the C1-C2 junction and load-deformation responses through simulated transoral odontoidectomy in human and baboon cadaveric specimens (Dickman et al., 1995). Our study also showed a similar result as Dickman et al. (Dickman et al., 1995) described and additionally demonstrated the instability of Oc-C1.

The C1-C2 junction is considered the main unstable segment after adenoidectomy (Dickman et al., 1992), and it is usually resolved with atlantoaxial fixation (Chang et al., 2016). However, the resection of the anterior C1 arch, anterior atlanto-occipital membrane, alar ligament, and apical ligament for odontoidectomy is related to instability of the occipital-C1. And in some cases, instability of the occipital-C1 already exists preoperatively, for example, in rheumatoid arthritis patients whose facet capsule ligament of occipital-C1 has been damaged by the primary disease. For these cases whose deformity or instability already exists at OC-C1 before odontoidectomy, the fixation of the occipital-C1 segment may be needed after odontoidectomy.

### Fixation techniques after odontoidectomy

Craniocervical fixation techniques have significantly advanced from wiring and cabling to fixation-based rigid segmental approaches in recent years. The development of internal fixation techniques has greatly enhanced the biomechanical stability of the CVJ and provided higher fusion rates. Posterior fixation is currently the most commonly used fixation technique for the instability of CVJ (Takayasu et al., 2016). However, posterior fixation techniques may be limited in special cases including severe thoracic kyphosis, congenital or medical defects of the posterior bony structures, and anomalous vertebral artery anatomy. When posterior fixation is not possible, a transarticular anterior approach fixation can be used as an alternative solution. This surgical approach can be performed concurrently with odontoidectomy, which avoids the additional trauma associated with a posterior approach (Dvorak et al., 2003b). In our study, the virtual implantation of AFF screws was performed according to the method described by Lu et al. (Lu et al., 1998), the junction of the lateral edge of the C2 vertebral body to 4 mm above the inferior edge of the C2 anterior arch was chosen for the starting point. In the later studies, other researchers made different improvements in AAF screw placement based on actual anatomy and surgical needs (Reindl et al., 2003; Sen et al., 2005; Wu et al., 2010). AAOF is a less studied technique than AAF, and only a few studies explored this procedure and its biomechanical properties. This technique was first introduced by Dvorak et al. (Dvorak et al., 2003a) in 2003, they performed the AAOF procedure in eight human cervical spine specimens and described the method of screw placement and safe extent. And in a follow up study (Dvorak et al., 2003b), they compared the biomechanical properties of this anterior screw technique with the traditional posterior fixation technique. After that, a few scholars further explored and improved this surgical method. Wu et al. (Wu et al., 2013) further explored the feasibility of percutaneous AAOF screw implantation and obtained good results in six patients. Moreover, according to the approach described by Dvorak et al., Cai et al. designed an anterior occiput-to-axis locking titanium plate system and explored the biomechanical performance differences between this technique and AAOF using finite element techniques (Cai et al., 2014). Besides rigid internal fixation, bone fusion also plays an important role in long-term postoperative stability (Goel, 2005; Goel et al., 2010). For transarticular screws, the most common method of bone grafting is posterior approach (Magerl and Seemann, 1987). Sasaki et, al (Sasaki et al., 2014) cured a patient with atlantoaxial instability by AAF and conventional posterior approach bone grafting. Dvorak et al. (Dvorak et al., 2003a) also presented in their article that the AAOF technique they invented required concomitant posterior bone grafting to facilitate long-term stable osseous union. However, Wu et al. (Wu et al., 2013) modified the AAOF procedure, they decorticated the front of the occiput-C2 region and C1/C2 articular process by curette and abrasive drilling after AAOF screws were implanted and then grafted iliac cancellous bone to the front of occiput-C2 region and C1/C2 articular process through the protective tube. In a word, compared with posterior fixation, there is less available experience for anterior fixation techniques, their specific procedure details including the position and length of the screws, and the bone grafting method require further exploration.

Given that internal fixation could provide rigid constructs to promote bony fusion and decrease the need for external immobilization, our study mainly investigated the biomechanical characteristics of internal fixations. In a human cervical spine specimen study, the stability of CVJ after transarticular anterior fixation has been proven to be comparable to that after posterior fixation techniques (Kim et al., 2004). Recently, due to the noninvasive and repeatable characteristics, finite element analysis was widely used to evaluate the biomechanical characteristic of different internal fixation techniques (Chun et al., 2018; Jain and Khan, 2021; Jain and Khan, 2022). However, the differences between anterior and posterior transarticular fixations in the FEM had not been investigated. Thus, we also compared the C1-C2 ROM values of our AAF and AAOF models with that of Chun et al.'s and Kim et al.'s posterior transarticular screws (PTS). The results (Supplementary Table S4) showed that AAF and AAOF models were comparable to PTS models in C1-C2 ROM values under extension + flexion. In addition, slightly larger C1-C2 ROM values were observed in AAF and AAOF than in Chun et al.‘s PTS model under lateral bending and torsion. The differences may be due to whole the cervical spine model used in our study, but the C1-C2 cervical spine for Chun et al.‘s study. The mean C1-C2 ROM values of Kim et al.‘s PTS screws in sixteen cervical spine specimens were significantly greater than that of AAF, AAOF, and Chun et al.‘s PTS FE models. This hints that there are some differences between cadaver and finite element studies.

AAOF and AAF were two classic anterior fixation techniques, and the biomechanical features of AAF after odontoidectomy had been demonstrated (Sen et al., 2005), but few studies had focused on the biomechanical characteristics of AAOF after odontoidectomy. To our knowledge, this is the first study to explore the biomechanical difference between AAF and AAOF after odontoidectomy. In the study, we simulated the biomechanical changes following anterior fixation of AAF and AAOF techniques after odontoidectomy based on a whole cervical spine model (-T1). Our results showed that AAOF and AAF produced similar decreases in ROM at the C1-C2 junction in extension, flexion, lateral bending, and torsion compared with the normal model (Figures 3C–F). Therefore, these two types of anterior fixations have the same effect in maintaining the stability of the C1-C2 segment. However, there were large differences in stability at Occiput-C1 between the AAOF and AAF fixation approaches. Due to additional occipital fixation, the movement of the Occiput-C1 segment can be significantly limited in all four directions after AAOF. In addition, ROM at Occiput-C1 significantly increased after AAF in all directions other than flexion. Therefore, we believe that AAOF is more effective in maintaining Occiput-C1 stability compared to AAF.

In our study, the maximum stress on the implant of AAOF was greater than that of AAF during extension and flexion. However, the differences can be ignored, as they were less than 10% (3.7% and 6.1% for extension and flexion, respectively) (the differences (%) of maximum stress on the implant between AAOF and AAF were calculated as (AAOF-AAF)/AAF). While the maximum stress on the implant of AAOF was significantly smaller than that of AAF during left bending, right bending, left torsion, and right torsion, the percent reductions for AAOF compared to AAF were 20.0%, 33.8%, 33.3%, and 24.9%, respectively. In addition, the maximum stress on the C2-C3 intervertebral disc did not significantly differ among the AAOF model, AAF model, and normal model.

### The feasibility and indications of using AAOF

Ideally, the AAOF implantation enters at the caudal of C2 and sequentially crosses the C1 lateral mass towards the occipital condyle (Figures 2G, K, L) (Dvorak et al., 2003a). The sublingual neural tube is located in the anterior-middle third of the occipital condyle and comprises important anatomical structures such as the sublingual nerve, the venous plexus, and the meningeal branch of the ascending pharyngeal artery (Gray et al., 1995). Based on the anatomy of the hypoglossal canal and the occipital condyle, it is relatively safe for the screw tip to be located at the posterolateral third of the occipital condyle. In addition, due to the entry point of AAOF which is far from the vertebroarterial foramen, the anterior approach is less likely to damage the vertebral artery. Given that this surgical procedure probably led to injury to important structures including hypoglossal nerves and vertebral arteries, comprehensive preoperative evaluation of the individual occipitoatlantoaxial joint anatomy preoperatively and intraoperative navigational device are required.

### Limitation

Although the material properties of the whole cervical spine model were derived from literature and the whole model had been validated by comparing it with previous results in our recent study. However, some deficiencies exist. Firstly, there is a certain gap between our FEM of the whole spine and the normal human body. The soft tissues such as muscles are ignored in this model, which has a certain influence on the experimental results. Besides, the shortcomings of not taking into account bone fusion in our study may lead to overestimated results in all simulations. The Finite element analysis is a physical approach to analyzing biomechanics, and once the bone fusion is modeled, there will be no mobility between the segments, so we cannot simulate the bone fusion using the finite element method. Thus, the computer-simulated results provided by Finite element analysis can only reflect the immediate postoperative status after internal fixation, rather than long-term stability.

## Conclusion

Overall, odontoidectomy can produce instability at the Occiput-C1, while it is not clear that the abnormally increased ROM at the Occiput-C1 requires further fixation. After odontoidectomy, the stability of the Occiput-C1 is primarily maintained by the atlanto-occipital joint. Thus, if the patients have a pre-existing injury to the atlanto-occipital joint before odontoidectomy, Occiput-C1 stability will further deteriorate after the operation. For example, patients with rheumatoid arthritis may have a pre-existing injury to the atlanto-occipital joint (Kandziora et al., 1999). Under these circumstances, the patients will probably require Occiput-C1 fixation, and AAOF is more suitable than AAF for these patients.

## Data Availability

The original contributions presented in the study are included in the article/Supplementary Material, further inquiries can be directed to the corresponding author.

## Abbreviations

AAOF, anterior axis-atlanto-occipital transarticular fixation; AAF, anterior atlantoaxial transarticular fixation; CVJ, craniovertebral junction; FEM, finite element model; ROM, range of motion.
