# Supplementary material for: Biomechanical comparison of anterior axis-atlanto-occipital transarticular fixation and anterior atlantoaxial transarticular fixation after odontoidectomy: A finite element analysis
Source: Front Bioeng Biotechnol. 2023 Mar 7;11:1067049. doi: 10.3389/fbioe.2023.1067049 (PMC10027935; doi:10.3389/fbioe.2023.1067049)
Supplement: Supplementary file 1 [file DataSheet1.pdf]

Supplementary Material

1 Supplementary Figures

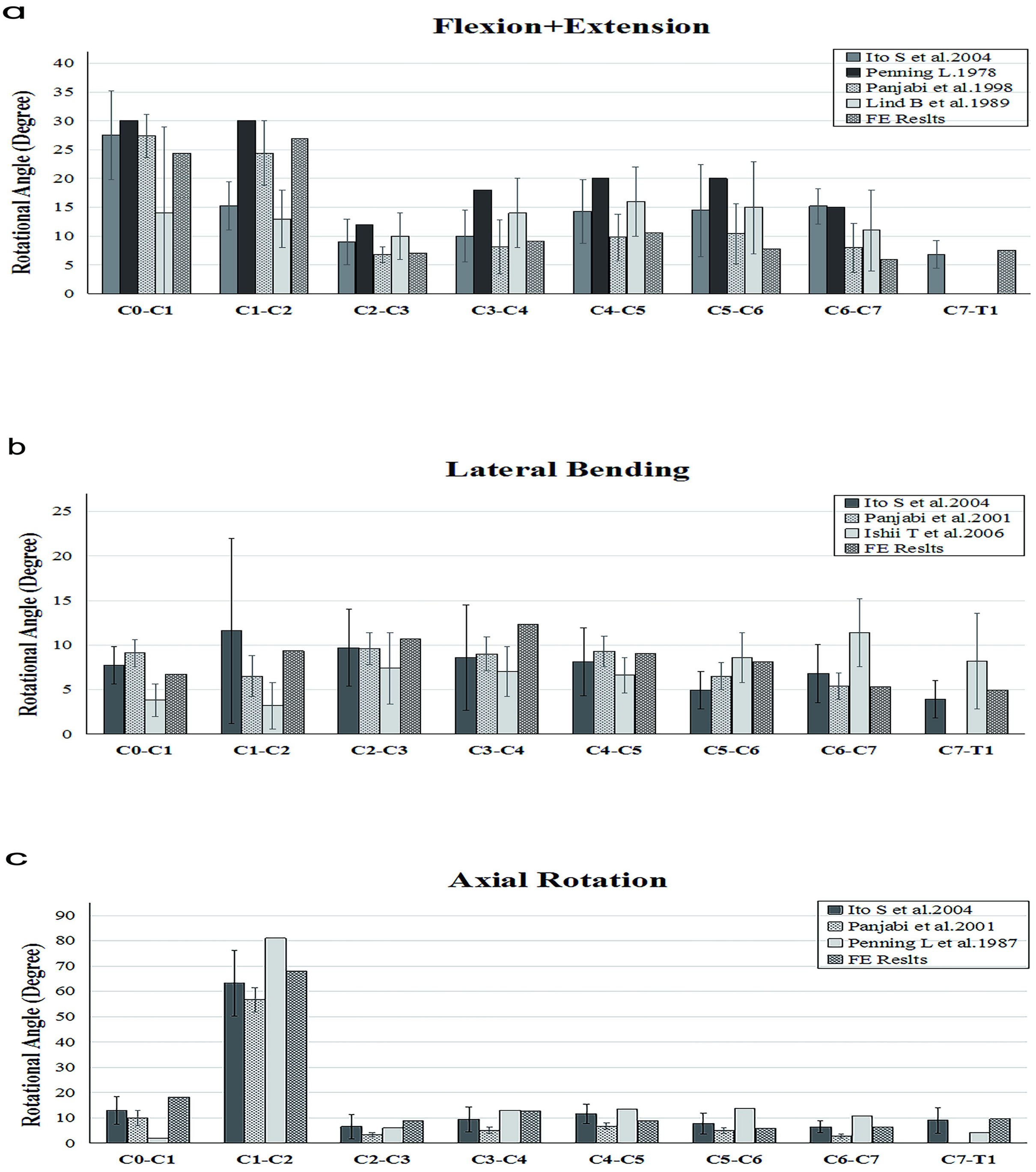

Supplementary Figure1. Comparison of the segmental rotation angle between the present FE model and the literature under flexion-extension (a)， lateral bending (b)， and axial rotation moments (c). Reproduced from Publication Clinical Neurology and Neurosurgery, 211, Tianhao Xie, Yu Feng, Bo Chen, Lianting Ma, Biomechanical evaluation of the craniovertebral junction after odontoidectomy with anterior C1 arch preservation: A finite element study, Pages 4-5, Copyright (2021), with permission from Elsevier.

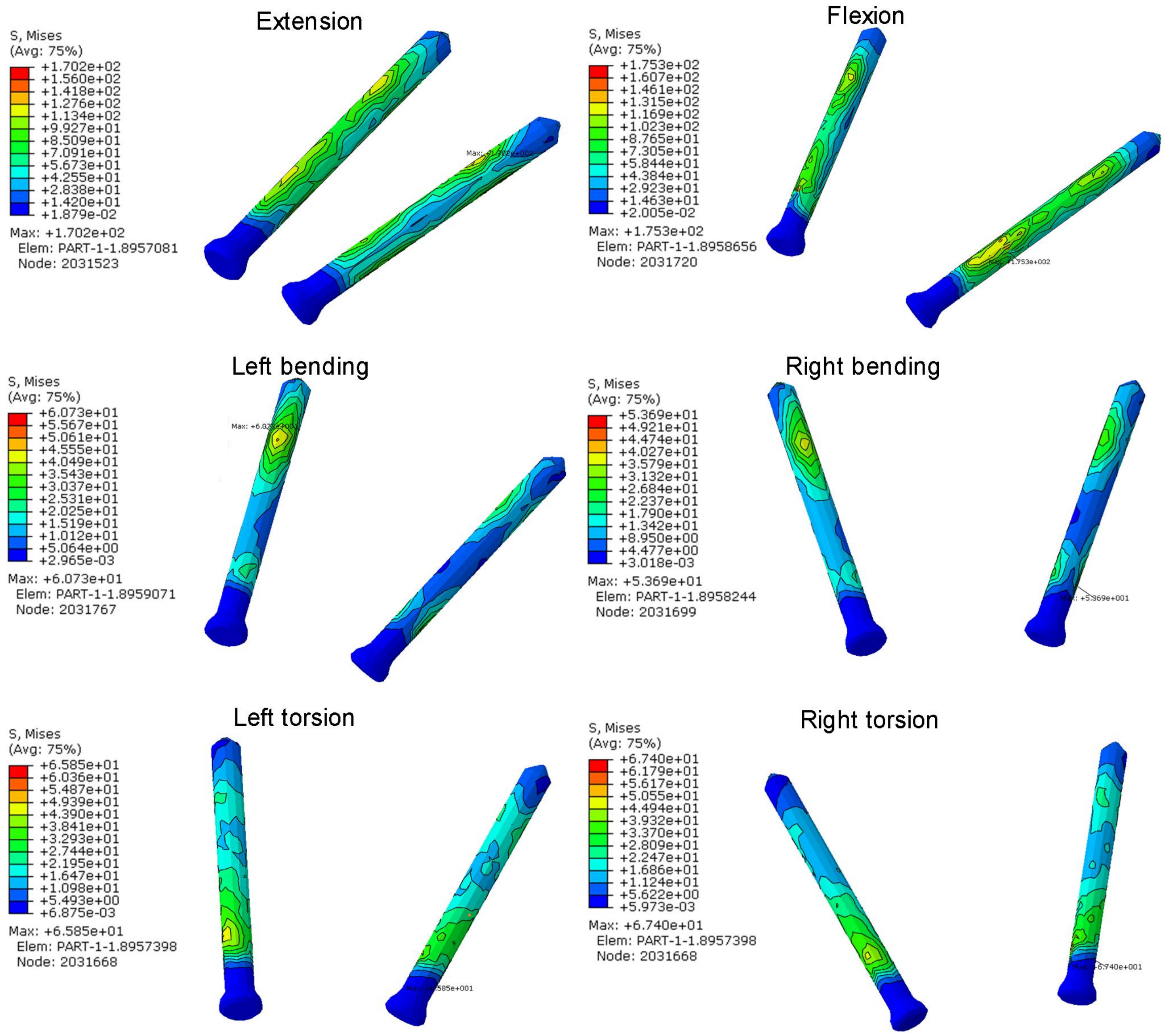

Supplementary Figure 2. Visualizations of the maximum von Mises stress for anterior axis-atlanto-occipital transarticular fixation in extension, flexion, left bending, right bending , left torsion, and right torsion.

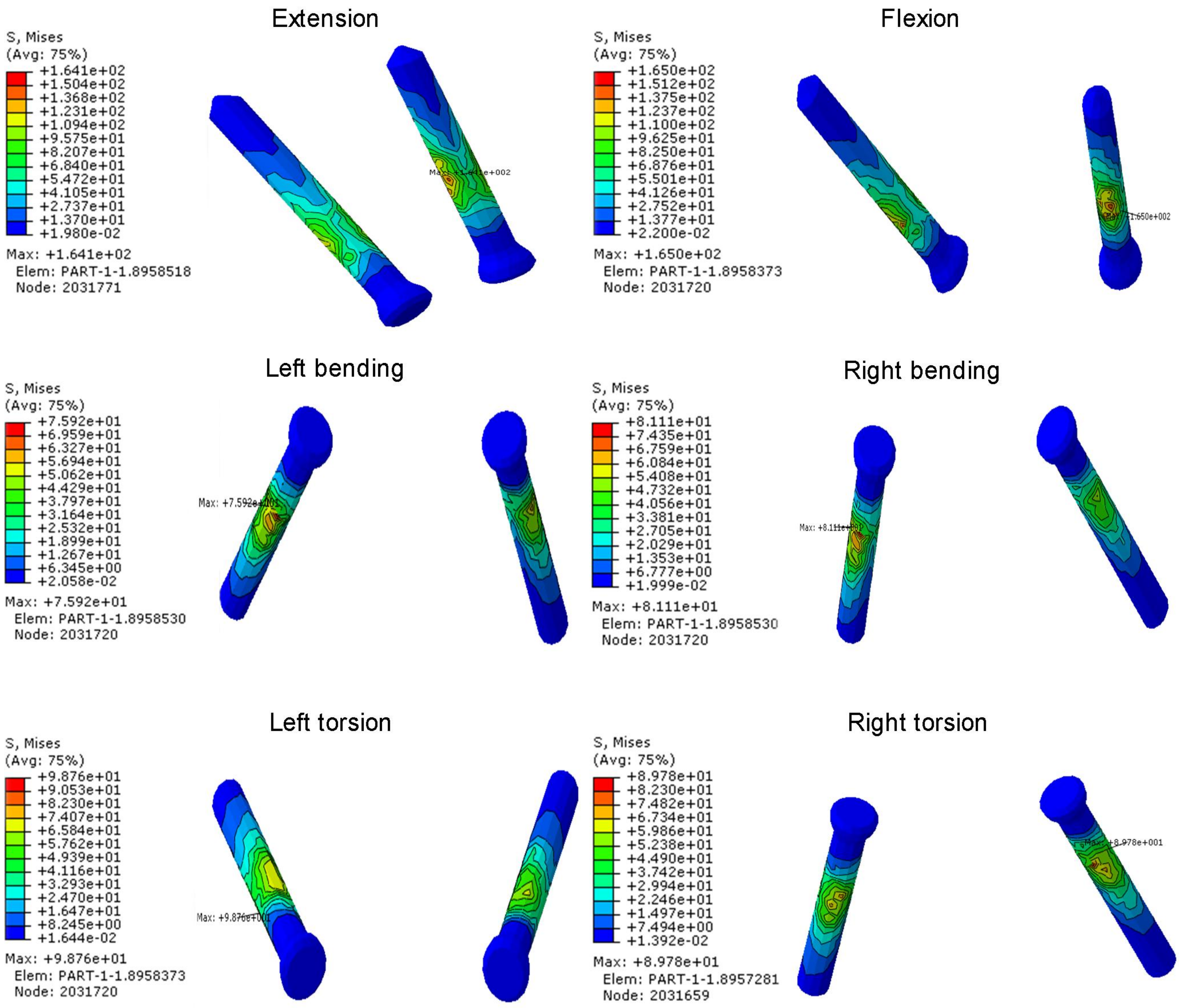

Supplementary Figure 3. Visualizations of the maximum von Mises stress for anterior atlantoaxial transarticular fixation in extension, flexion, left bending, right bending, left torsion, and right torsion.

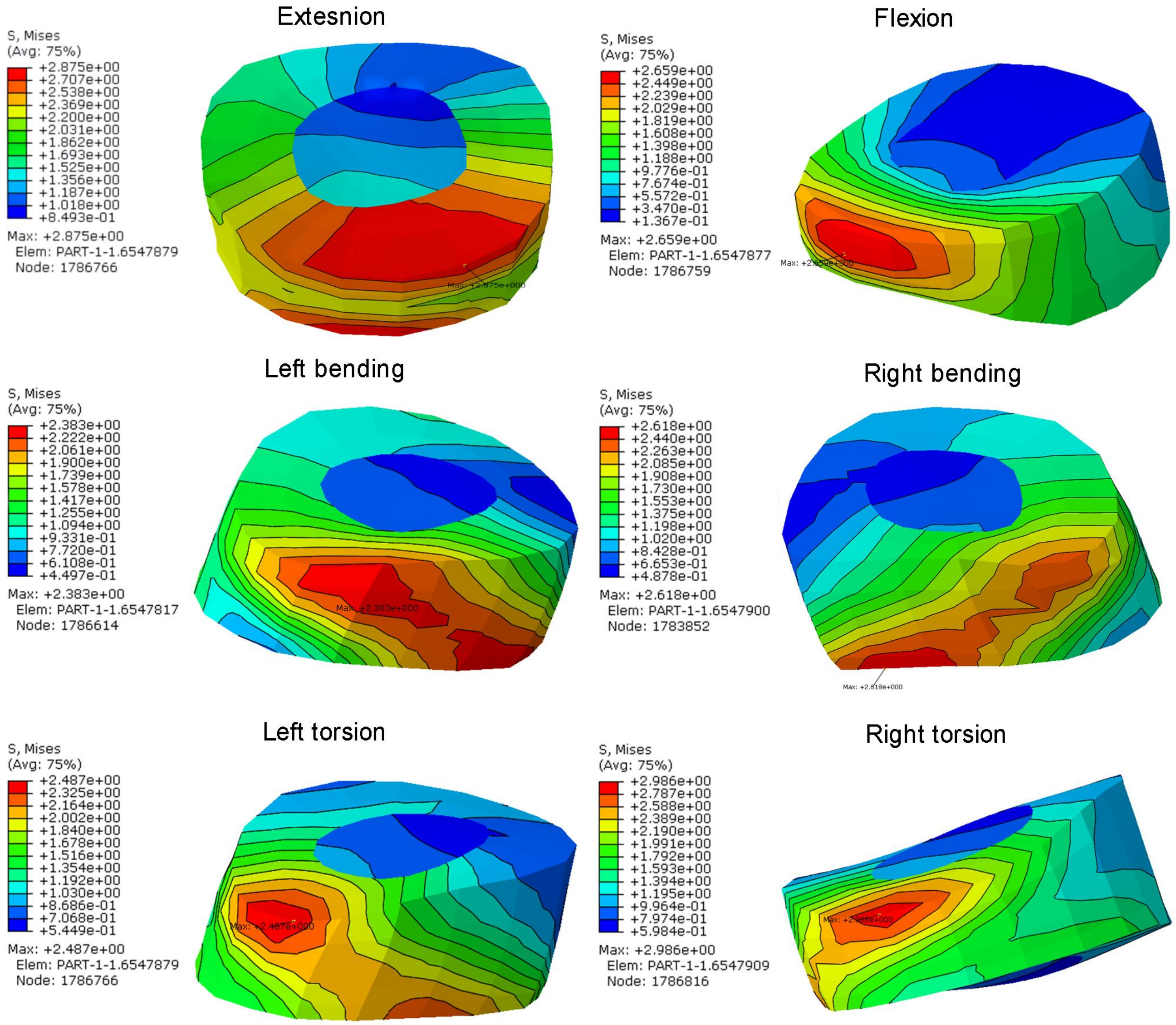
Extesnion
S, Mises
(Avg: 75%)
+2.875e+00
+2.707e+00
+2.538e+00
+2.369e+00
+2.200e+00
+2.031e+00
+1.862e+00
+1.693e+00
+1.525e+00
+1.356e+00
+1.187e+00
+1.018e+00
+8.493e-01
Max: +2.875e+00
Elem: PART-1-1.6547879
Node: 1786766
Flexion
S, Mises
(Avg: 75%)
+2.659e+00
+2.449e+00
+2.239e+00
+2.029e+00
+1.819e+00
+1.608e+00
+1.398e+00
+1.188e+00
+9.776e-01
+7.674e-01
+5.572e-01
+3.470e-01
+1.367e-01
Max: +2.659e+00
Elem: PART-1-1.6547877
Node: 1786759
Left bending
S, Mises
(Avg: 75%)
+2.383e+00
+2.222e+00
+2.061e+00
+1.900e+00
+1.739e+00
+1.578e+00
+1.417e+00
+1.255e+00
+1.094e+00
+9.331e-01
+7.720e-01
+6.108e-01
+4.497e-01
Max: +2.383e+00
Elem: PART-1-1.6547817
Node: 1786614
Right bending
S, Mises
(Avg: 75%)
+2.618e+00
+2.440e+00
+2.263e+00
+2.085e+00
+1.908e+00
+1.730e+00
+1.553e+00
+1.375e+00
+1.198e+00
+1.020e+00
+8.428e-01
+6.653e-01
+4.878e-01
Max: +2.618e+00
Elem: PART-1-1.6547900
Node: 1783852
Left torsion
S, Mises
(Avg: 75%)
+2.487e+00
+2.325e+00
+2.164e+00
+2.002e+00
+1.840e+00
+1.678e+00
+1.516e+00
+1.354e+00
+1.192e+00
+1.030e+00
+8.686e-01
+7.068e-01
+5.449e-01
Max: +2.487e+00
Elem: PART-1-1.6547879
Node: 1786766
Right torsion
S, Mises
(Avg: 75%)
+2.986e+00
+2.787e+00
+2.588e+00
+2.389e+00
+2.190e+00
+1.991e+00
+1.792e+00
+1.593e+00
+1.394e+00
+1.195e+00
+9.964e-01
+7.974e-01
+5.984e-01
Max: +2.986e+00
Elem: PART-1-1.6547909
Node: 1786816

Supplementary Figure 4. Visualizations of the maximum von Mises stress on the C2-C3 disc for anterior axis-atlanto-occipital transarticular fixation in extension, flexion, left bending, right bending, left torsion, and right torsion.

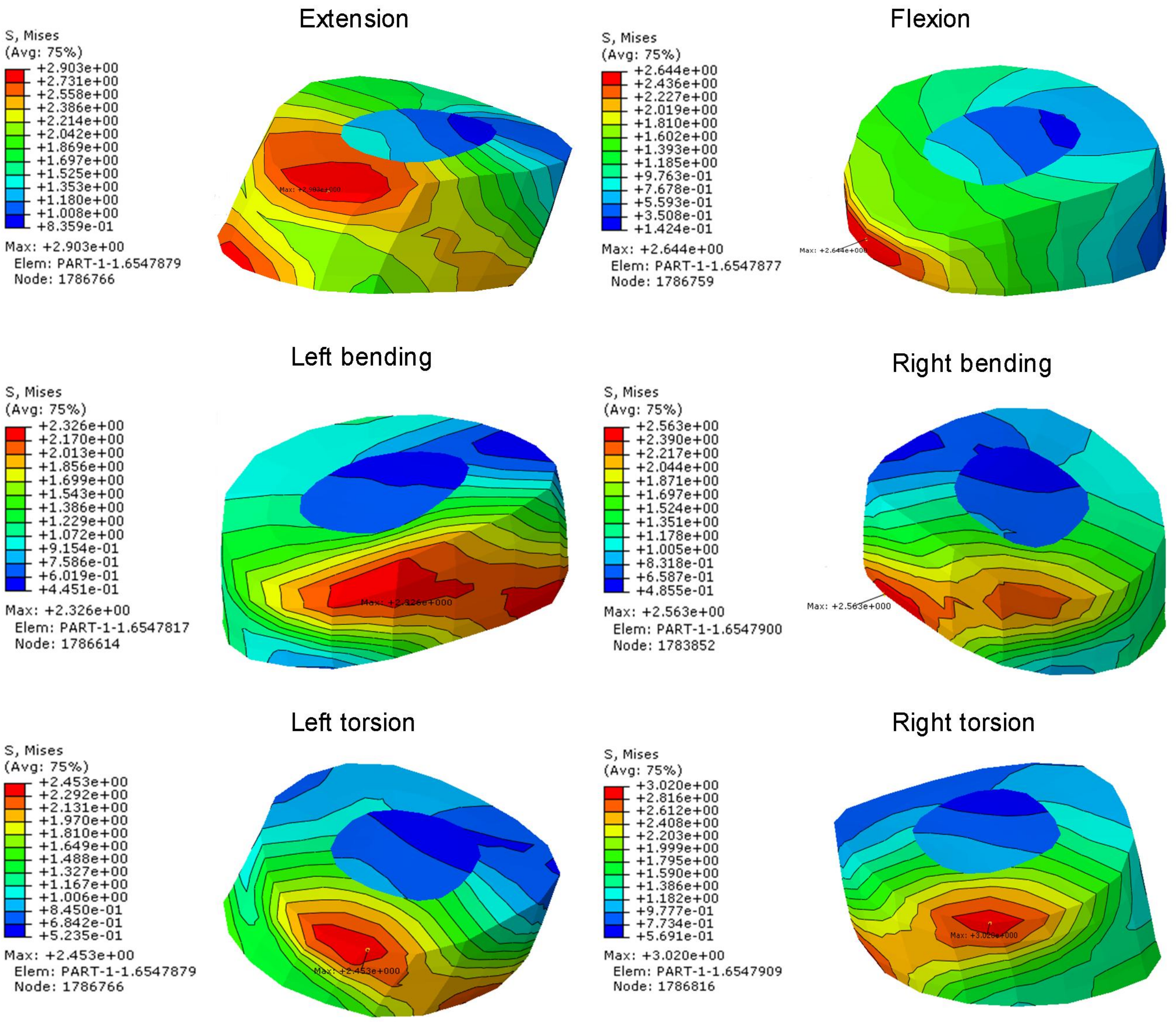

Supplementary Figure 5. Visualizations of the maximum von Mises stress on the C2-C3 disc for anterior atlantoaxial transarticular fixation in extension, flexion, left bending, right bending, left torsion, and right torsion.

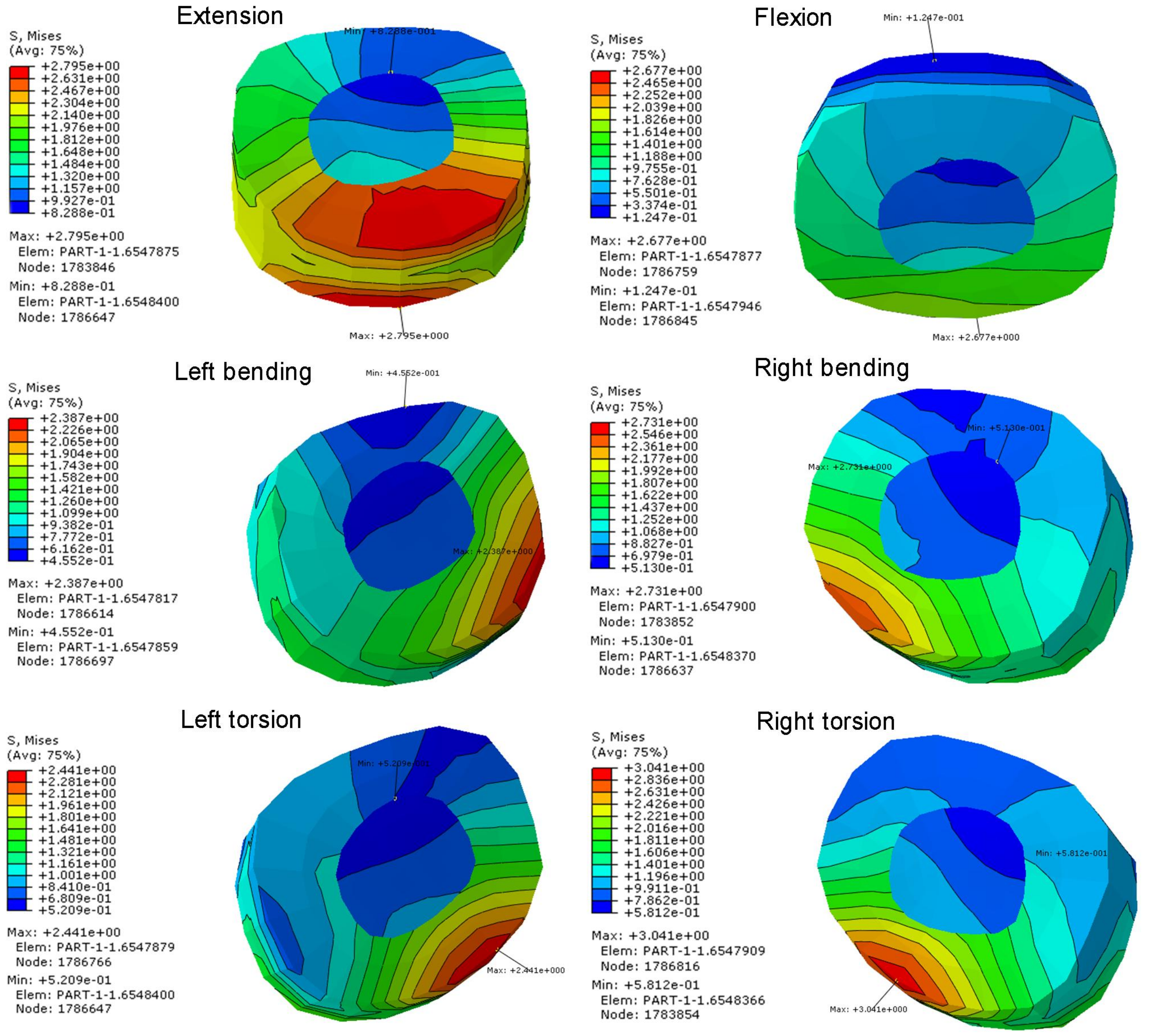

Supplementary Figure 6. Visualizations of the maximum von Mises stress on the C2-C3 disc for the normal model in extension, flexion, left bending, right bending, left torsion, and right torsion.

1.1 Supplementary Tables

Supplementary Table 1. Material Property for the Cervical Spine Finite Element Model (Yoganandan et al., 2001; Brolin and Halldin, 2004; Zhang et al., 2006; Zhang and Bai, 2007; Panzer et al., 2011; Xie et al., 2021).

Description	Element Type	Young's	Poisson's

		Modulus (MPa)	Ratio
Cortical bone	3-D solid (6 node) (trihedral)	10000	0.29
Cancellous bone	3-D solid (4 node) (tetrahedral)	100	0.29
Posterior elements	3-D solid (4 node) (tetrahedral)	3500	0.4
Endplate	3-D solid (6 node) (trihedral)	500	0.29
Annulus ground substance	3-D solid (8 node) (hexahedral)	3.4	0.4
Annulus fibrosus	Tension only linear contact elements (2 node)	450	0.3
Disc–nucleus	3-D solid (8 node) (hexahedral)	1	0.499

Reproduced from Publication Clinical Neurology and Neurosurgery, 211, Tianhao Xie, Yu Feng, Bo Chen, Lianting Ma, Biomechanical evaluation of the craniovertebral junction after odontoidectomy with anterior C1 arch preservation: A finite element study, Page 2, Copyright (2021), with permission from Elsevier.

Reference

Brolin, K., and Halldin, P. (2004). Development of a finite element model of the upper cervical spine and a parameter study of ligament characteristics. Spine (Phila Pa 1976) 29(4), 376-385.http://dx.doi.org/10.1097/01.brs.0000090820.99182.2d

Panzer, M.B., Fice, J.B., and Cronin, D.S. (2011). Cervical spine response in frontal crash. Med Eng Phys 33(9), 1147-1159.http://dx.doi.org/10.1016/j.medengphy.2011.05.004

Yoganandan, N., Kumaresan, S., and Pintar, F.A. (2001). Biomechanics of the cervical spine Part 2. Cervical spine soft tissue responses and biomechanical modeling. Clin Biomech (Bristol, Avon) 16(1), 1-27.http://dx.doi.org/10.1016/s0268-0033(00)00074-7

Zhang, H., and Bai, J. (2007). Development and validation of a finite element model of the occipito-atlantoaxial complex under physiologic loads. Spine (Phila Pa 1976) 32(9), 968-974.http://dx.doi.org/10.1097/01.brs.0000261036.04919.91

Zhang, Q.H., Teo, E.C., Ng, H.W., and Lee, V.S. (2006). Finite element analysis of moment-rotation relationships for human cervical spine. J Biomech 39(1), 189-193.http://dx.doi.org/10.1016/j.jbiomech.2004.10.029

Supplementary Table 2. Material properties of the spinal Ligaments at C0-C2

Description	Segment	d_f (mm)	f_f (N)	f_n:f_f	d_n:d_f
Facet capsule ligament	C0-C1	9.9	320	1:10	1:3
Anterior atlantooccipital membrane	C0–C1	18.9	232	1:10	1:5
Posterior atlantooccipital membrane	C0–C1	18.1	83	1:10	1:3
Anterior longitudinal ligament	C1–C2	11.8	263	1:10	1:5
Facet capsule ligament	C1–C2	9.3	314	1:10	1:2
Ligamentum flavum	C1–C2	9.6	111	1:10	1:3
Tectorial membrane	C0–C2	11.9	76	1:10	1:3
Apical ligament	C0–C2	8.0	214	1:10	1:5
Alar ligament	C0–C2	14.1	357	1:10	1:5
Cruciate ligament vertical portion	C0–C2	12.5	436	1:10	1:5

Reproduced from Publication Clinical Neurology and Neurosurgery, 211, Tianhao Xie, Yu Feng, Bo Chen, Lianting Ma, Biomechanical evaluation of the craniovertebral junction after odontoidectomy with anterior C1 arch preservation: A finite element study, Page 2, Copyright (2021), with permission from Elsevier.

d_n=The length of the neutral zone, d_f=The length of the failure zone

f_f=The force of the failure zone, f_n=The force of the neutral zone

Supplementary Table 3. Material properties of the spinal Ligaments at C2-T1

Anterior	Posterior	Spinous	Ligamentum	Capsular

Longitudinal		Longitudinal				Flavum			
Deflection (mm)	Force (N)	Deflection (mm)	Force (N)	Deflection (mm)	Force (N)	Deflection (mm)	Force (N)	Deflection (mm)	Force (N)
C2–C5									
-10	0	-10	0	-10	0	-10	0	-10	0
-1	0	-1	0	-1	0	-1	0	-1	0
0	0	0	0	0	0	0	0	0	0
1	28	1	25	1	7	2	38	2	55
2	52	2	44	2	12.5	4	60	4	130
3	72	3	62	3	18	6	80	6	180
4	89	4	78	4	22.5	8	108	8	210
5	102	5	89	5	26			10	230
6	115			6	30				
				7	32.5				
C5–T1									
-10	0	-10	0	-10	0	-10	0	-10	0
-1	0	-1	0	-1	0	-1	0	-1	0
0	0	0	0	0	0	0	0	0	0
1	20	1	20	1	8	2	30	2	75
2	40	2	40	2	14	4	68	4	145
3	58	3	60	3	20	6	102	6	204
4	78	4	78	4	25	8	130	8	250
5	98	5	92	5	29	10	145	10	265
6	112			6	32.5				

7	120	7	35

Supplementary Table 3 was reproduced from Publication Clinical Neurology and Neurosurgery, 211, Tianhao Xie, Yu Feng, Bo Chen, Lianting Ma, Biomechanical evaluation of the craniovertebral junction after odontoidectomy with anterior C1 arch preservation: A finite element study, Page 3, Copyright (2021), with permission from Elsevier.

Supplementary Table 4. Comparison among AAF, AAOF, and PTS (Kim et al., 2004; Chun et al., 2018)

C1-C2 (ROM)	AAOF	AAF	PTS (Chun et.al)	PTS (Kim et.al)
Extension+Flexion	4.62	4.67	4.40	17.10
Lateral bending	1.70	1.54	0.01	13.90
Torsion	5.80	7.34	0.35	48.10

AAOF=anterior axis-atlanto-occipital transarticular fixation; AAF=anterior atlantoaxial transarticular fixation;PTS=posterior transarticular screw.

Chun, D.H., Kim, K.N., Yi, S., Shin, D.A., and Ha, Y. (2018). Biomechanical comparison of four different atlantoaxial posterior fixation constructs in adults: a finite element study. *Spine* 43(15)**,** E891-E897

Kim, S.-M., Lim, T.J., Paterno, J., Hwang, T.-J., Lee, K.-W., Balabhadra, R.S., et al. (2004). Biomechanical comparison of anterior and posterior stabilization methods in atlantoaxial instability. *Journal of Neurosurgery: Spine* 100(3)**,** 277-283
